# Enhanced Recovery after Surgery (ERAS) Protocol Is a Safe and Effective Approach in Patients with Gastrointestinal Fistulas Undergoing Reconstruction: Results from a Prospective Study

**DOI:** 10.3390/nu13061953

**Published:** 2021-06-07

**Authors:** Stanislaw Klek, Jerzy Salowka, Ryszard Choruz, Tomasz Cegielny, Joanna Welanyk, Mariusz Wilczek, Kinga Szczepanek, Magdalena Pisarska-Adamczyk, Michal Pedziwiatr

**Affiliations:** 1 Surgical Oncology Clinic, National Cancer Institute, 31-115 Krakow, Poland; jo.garbacik@wp.pl (J.W.); msmariow@wp.pl (M.W.); 2 General Surgery Unit with Intestinal Failure Center, Stanley Dudrick’s Memorial Hospital, 32-082 Skawina, Poland; jsalowka@gmail.com (J.S.); rchoruz@poczta.fm (R.C.); tcegielny@op.pl (T.C.); kingaszm@interia.pl (K.S.); 3 Department of Medical Education, Jagiellonian University Medical College, 30-688 Krakow, Poland; magdalenapisarska@interia.pl; 4 2nd Department of General Surgery, Jagiellonian University Medical College, Jakubowskiego 2, 30-688 Kraków, Poland; mpedziwiatr@googlemail.com

**Keywords:** ERAS, gastrointestinal surgery, GI tract, reconstruction

## Abstract

Background and Aims: An enterocutaneous fistula (ECF) poses a major surgical problem. The definitive surgical repair of persistent fistulas remains a surgical challenge with a high rate of re-fistulation and mortality, and the reasons for that is not the surgical technique alone. Enhanced Recovery after Surgery (ERAS^®^) is an evidence-based multimodal perioperative protocol proven to reduce postoperative complications. The aim of the study was to assess the clinical value of the ERAS protocol in surgical patients with ECF. Methods: ERAS protocol was used in all patients scheduled for surgery for ECF at the Stanley Dudrick’s Memorial Hospital in Skawina between 2011 and 2020. A multidisciplinary team (MDT) was in charge of the program and performed annual audits. A consecutive series of 100 ECF patients (44 females, 56 males, mean age 54.1 years) were evaluated. Postoperative complications rate, readmission rate, length of hospital stay, prevalence of postoperative nausea and vomiting were assessed. Registered under ClinicalTrials.gov Identifier no. NCT04771832. Results: ERAS protocol was successfully introduced for ECF surgeries; however, eight modifications to the ERAS program was performed in 2015. They led to improvement of surgical outcomes: reduction of postoperative nausea and vomiting (15 vs. 17% patients, *p* = 0.025), overall complication rate (11 vs. 10, *p* = 0.021), median length of hospital stay (overall and after surgery, *p* = 0.022 and 0.002, respectively). Conclusions: ERAS protocol can be successfully used for ECF patients. Prescheduled audits can contribute to the improvement of care.

## 1. Introduction

An enterocutaneous fistula (ECF) is an abnormal connection (fistula) between the intestine and the skin. It can develop spontaneously, as a complication of the inflammatory bowel disease or radiotherapy, but most often develops postoperatively as a result of iatrogenic intestinal lesions or leaking anastomosis [1]. The incidence of ECF has been estimated to be below 0.5 patients per 100,000 inhabitants and thought to complicate 0.8% to 2% of abdominal operations; it is one of the orphan diseases [2,3].

The treatment of patients with an ECF can be challenging and unsatisfactory, as the mortality rate can reach up to 10% [4,5]. It is mainly the consequence of sepsis, malnutrition, and electrolyte imbalances [4,6]. Surgery comes as the last step of the treatment, when the spontaneous closure is impossible. Generally speaking, 60% to 80% of patients will usually require a restorative procedure which is successful in 85% to 90% of these cases [7,8].

The definitive surgical repair of persistent fistulas remains a surgical challenge with a high rate of re-fistulation and mortality, and the reasons for that is not the surgical technique alone [5]. Another important issue is the optimal perioperative care. Introduced over a decade ago, Enhanced Recovery after Surgery (ERAS^®^) is an evidence-based multimodal perioperative protocol focused on stress reduction and the promotion of a return to function [9]. Evidence from both observational and RCTs supports reduced morbidity with the implementation of ERAS, including reduction in specific postoperative complications like surgical site and urinary tract infections. Surprisingly, a study on the implementation of ERAS in surgery for ECF has never been published.

The aim of the study was to assess the clinical value of ERAS protocol in surgical patients with ECF.

## 2. Methods

The study was performed at the General and Cancer Surgery Unit with the Intestinal Failure Center of the Stanley Dudrick’s Memorial Hospital in Skawina, Poland. Starting January 2011, ERAS protocol was used in all patients scheduled for surgery for ECF.

The following components of ERAS protocol were implemented:

(a) Preoperative: pre-admission education (health education, exercise advice, dietary guidance), organ function evaluation, minimized preoperative fasting (Fasting from solid food for 6 h and drinking ad libidum for 2 h before operation), carbohydrate loading, no or selective bowel preparation, venous thromboembolism prophylaxis and intravenous antibiotic prophylaxis.
(b) Intraoperative: intraoperative safety check (WHO check list), active warming, opioid-sparing analgesia, including preemptive analgesia (acetaminophen), thoracic epidural analgesia (TEA) in case of laparotomy, precision surgery scheme, minimally invasive surgical techniques if available, avoidance prophylactic NG tubes and drains, no indwelling nasogastric tube, near-zero fluid balance, postoperative nausea and vomiting (PONV) prophylaxis.
(c) Postoperative: early oral nutrition, mobilization on the first postoperative day, early catheter removal, early extraction of abdominal drainage tube (<48), near-zero perioperative fluid balance fluid management, pain and nausea management.

All aspects were presented in Table 1.

The multidisciplinary team (MDT), composed of two surgeons, two anesthetists, two surgical and one anesthesia nurse, physiotherapist, dietitian and psychologist, was established in January 2011 and made responsible for supervising the ERAS protocol. 

MDT decided to implement all components of ERAS from the very beginning of the center’s activity and re-evaluate the policy every 12 months. Modifications of the policy were allowed if the majority of MDT (>50%) voted for the change. 

The consecutive series of a hundred patients was selected as the target group eligible for evaluation of the effectiveness and safety of the protocol.

Modifications of the protocol were supposed to reduce complications and/or compliance. Any aspect of each ERAS component could be verified and modified in any terms, including drug type, dose, procedure or intervention.

To evaluate the treatment efficacy following aspects were measured and compared at the beginning (January 2011) and the end (December 2020) of the observation period:

- Postoperative complications;
- Length of hospital stay (total and after surgery);
- Prevalence of postoperative nausea and vomiting;
- Time to first flatus;
- Readmission rates.

To achieve this, patients were divided into two major groups: group 1 was formed of patients operated on between 2011 and 2015, and group 2 was formed of patients undergoing surgery between 2016 and 2020.

## 3. Statistics

All data were analyzed with Statsoft STATISTICA v.13 (StatSoft Inc., Tulsa, OK, USA). A descriptive study of the sample was carried out. Numerical variables are presented as mean ± standard deviation (SD) or median with interquartile range (IQR) if the distributions were nonparametric. The Pearson chi-square test of independence was used to examine the relationship between each variable and outcome. Fisher’s exact test was used when the conditions for the chi 2 test were not met. The Shapiro–Wilk test was used to check for normal distribution of data and the T-student test was used for normally distributed quantitative data. For non-normally distributed quantitative variables, the Mann–Whitney U test was used. A *p*-value < 0.05 was considered statistically significant.

All procedures were performed in accordance with the ethical standards laid down in the 1964 Declaration of Helsinki and its later amendments. Informed consent for proposed surgical treatment was obtained from all patients before surgery. This study was approved by the institutional research ethics board of National Cancer Institute in Krakow (KBET 27/10/2020) and was registered in the ClinicalTrials.gov.

## 4. Results

One hundred patients (44 females, 56 males, mean age 54.1 years) were operated on for gastrointestinal fistula and the restoration of the GI tract continuity was achieved. Patient profile was presented in Table 2.

Components of ERAS protocol were evaluated every 12 months. No significant changes were made in 2012, 2013, 2014, 2017, 2018, or 2019.

Table 3 presents modifications to the protocol. 

Modifications of the protocol in 2015 led to improvement of surgical outcomes: reduction of postoperative nausea and vomiting (15 vs. 17 patients, *p* = 0.025), overall complication rate (11 vs. 10, *p* = 0.021), median length of hospital stay (overall and after surgery, *p* = 0.022 and 0.002, respectively). Complications other than those mentioned above that were evaluated included: surgical site infection, cardiopulmonary complications, urinary tract infections, anastomosis leak, abdominal wall dehiscence, intrabdominal fluid or abscess, collection, intra-abdominal bleeding, and postoperative paralytic ileus. Table 4 presents detailed characteristics of treatment outcomes. 

## 5. Discussion

Surgery for ECF can be successful, yet demanding. In the Dutch center study, overall closure was achieved in 118 patients (87.4%) and restorative operations were successful in 97/107 patients (90.7%) [4]. Unfortunately, ECF surgical patients quite frequently develop complications. In Visschers’ study, mortality rate reached 9.6% [4] and Klucinski et al. showed that severe complications (Clavien–Dindo grade III–V) made up 28.0% of all complications [5]. The fistula complexity determines the risk of severe postoperative complications or fistula recurrence after definitive surgical repair [4,5]. The high prevalence of postoperative complications if EFC patients should not be surprising, even in elective colorectal surgery the incidence of postoperative nausea and vomiting reaches (25–40%) [10].

Hence, the need for an improvement, and ERAS protocol seems to be a perfect solution to the problem. In colorectal surgery, ERAS protocol is already well established as the best care [11], because it has been proven to lower both recovery time and postoperative complication rates while being cost-effective at the same time [12] ERAS guidelines are now available for almost every type of major surgery, including colorectal, gastric, liver, pancreatic, esophageal, cytoreductive, cardiac, bariatric, lung, breast, and total hip/knee replacement. 

In 2011, immediately after opening the surgical center in Skawina, ERAS protocol was introduced at our center for all types of major gastrointestinal procedures. Unlike for cancer surgeries, the necessity for modifications of initial recommendations was expected. Therefore, an internal auditing system was established. Annual meetings led to significant modifications of components of perioperative care, and the latter to the improvement of outcomes.

As expected, postoperative nausea and vomiting (PONV) was one of the most common issues. The use of the chewing gum and morning coffee, introduced from the early beginning, and early oral feeding led to the PONV prevalence of 46.9%. A change in the protocol, which was the allowance of liquids POD 1 instead of solid meals, helped to reduce PONV to 24.6%. No coffee or chewing gum was used.

Another revision, which was using single draining tube in case of large space in the abdominal cavity, helped to reduce the surgical complication rate from 34.4% to 14.5%. All protocol modifications from the 2015 MDT meeting also led to shortening of the length of hospital stay (overall and after surgery, *p* = 0.022 and 0.002, respectively).

## 6. Conclusions

To our knowledge, this is the first study on the implementation of ERAS in surgery for ECF. It showed that enhanced recovery program can be successfully used even for major, potentially risky surgery. It also demonstrated that audits are inevitable part of modern perioperative care, as constant modifications can contribute to the improvement of care.

## Figures and Tables

**Table 1:** Presents each component of the ERAS protocol implemented in January 2011.

Name of the Component	Detailed Description
Preoperative	
Pre-admission education (health education, exercise advice, dietary guidance)	Conversation between surgeon and anesthetist and a patient
Organ function evaluation	Lab tests including erythrocytes count and HbA1c
Minimized preoperative fasting	Patient allowed to consume low residual diet up to 6 h before surgery, 800 mL of 12.5% Maltodextrine-containing drink in the afternoon and evening day before surgery
Carbohydrate loading	400 mL of 12.5% Maltodextrine-containing drink up to 2 h before operation
No or selective bowel prep	Two rectal enemas (in the evening of the day before and in the morning of the day of surgery
Venous thromboembolism prophylaxis	Low molecular weight heparine
Antibiotic prophylaxis	Surgical site infection prophylaxis only: cefazoline + metronidazole 30–60 min before surgery
Intraoperative	
Active warming	Bair-hugger, deep temperature measurement
Anesthesia	Propofol for induction combined with short acting opioids. Short acting inhalational agents in oxygen enriched mixture
Analgesia opioid-sparing multimodal technique	Preemptive acetominophen, TEA ***, lidocaine infusion, NSAIDs *
Minimally invasive surgical techniques if available	Laparoscopy, reduction of incision size, transverse incisions
Avoidance prophylactic NG tubes	No tube during surgery
Avoidance prophylactic drains	No drains
Near-zero perioperative fluid balance	4 h urinary output measurement
PONV prophylaxis	Dexamethazone, metoclopramide, ondansetron
Postoperative	
Early oral nutrition	Drinking and solid food allowed on POD 1 **
Mobilization on the first postoperative day	Full mobilization from POD 1 **
Early catheter removal	Removal of the catheter on POD 1
Early extraction of abdominal drainage tube (<48 h)	No drainage
Near-zero fluid balance	Intravenous fluids reduced to below 1000 mL per day, patient’s weight every day
Pain management	Acetaminophen, NSAIDs, TEA ***

* NSAIDs—non steroid anti-inflammatory drug. ** POD—postoperative day. *** Thoracic epidural anesthesia.

**Table 2:** Demographic analysis of patients.

Parameter	2011–2015	2016–2020	*p* Value
Number of patients, *n*	32	69	-
Females, *n* (%)	11 (34.4%)	33 (47.8%)	0.205
Males, *n* (%)	21 (65.6%)	36 (52.2%)	
Mean age, years ± SD	53.9 ± 14.5	55.9 ± 14.1	0.514
Mean HbA1 concentration	2.3 ± 2.1	2.7 ± 1.9	0.614
Mean Hemoglobin	13.4 ±6.2	13.5 ± 5.7	0.701
Anastomosis, *n* (%)			0.665
small intestine + small intestine	18 (56.3%)	45 (65.2%)	
small intestine + colon	7 (21.9%)	13 (18.8%)	
colon + colon	7 (21.9%)	11 (15.9%)	
Underlying (primary) disease, *n* (%)			
Actinomycosis	1 (3.1%)	-	
Adhesion	1 (3.1%)	2 (2.9%)	
Cancer	7 (21.9%)	36 (52.2%)	
Ulcerative colitis	2 (6.3%)	2 (2.9%)	
Diverticulitis	3 (9.4%)	1 (1.4%)	
Bowel ischemia	8 (25%)	15 (21.7%)	
Crohn’s diseases	8 (25%)	13 (18.8%)	
Pressure ulcer	2 (6.3%)	-	

**Table 3:** Modifications to the protocol.

Name of the Component	Modification	
	2015	2016
Preoperative		
Pre-admission education (health education, exercise advice, dietary guidance)	No change	Printed booklets
Organ function evaluation	No change	CEA and Ca 19–9 introduced as a part of lab testing
Minimized preoperative fasting	800 mL of 12.5% Maltodextrin-containing drink—terminated, patients allowed to consume low residual day before surgery	No change
Carbohydrate loading	No change	No change
No or selective bowel prep	Osmotic agent (one dose per day) recommended for 3 days before operation if protective ileostomy to be performed during anastomosis to the rectum	No change
Venous thromboembolism prophylaxis	No change	no change
Antibiotic prophylaxis	No change	No change
Intraoperative		
Active warming	No change	No change
Opioid-sparing technique	No change	No change
Minimally invasive surgical techniques if available	No change	No change
Avoidance prophylactic NG tubes	No change	No change
Avoidance prophylactic drains	One draining tube to be inserted in case of large space in the abdominal cavity	No change
Goal directed peri-operative fluid management	No change	No change
Pain and nausea management	Metamizole introduced as a part of analgesia	No change
Postoperative		
Early oral nutrition	Oral nutritional supplements and clear drinks without solid food on POD 1	No change
Mobilization on the first postoperative day	No change	No change
Early catheter removal	Allowed removal on POD 2 or 3 in case of poor mobilization or rectal surgery	No change
Early extraction of abdominal drainage tube (<48 h)	Introduction of that policy	No change
Near-zero fluid balance	No change	No change
Pain management	Metamizole and TAP block * introduced as a part of analgesia, lidocaine infusion during laparoscopic surgery	No change

* transversus abdominis plane block.

**Table 4:** Postoperative outcomes in analyzed groups.

Parameter	Group 1	Group 2	*p* Value
Postoperative nausea and vomiting, *n* (%)	15 (46.9%)	17 (24.6%)	0.025
Median Time to first flatus, days (IQR)	3 (2–5)	2 (2–3)	0.204
Patients with complications, *n* (%)	11 (34.4%)	10 (14.5%)	0.021
Clavien–Dindo 1, *n* (%)	3 (9.6%)	2 (2.8%)	0.859
Clavien–Dindo 2, *n* (%)	2 (6.2%)	2 (2.8%)	
Clavien–Dindo 3, *n* (%)	2 (6.3%)	3 (4.3%)	
Clavien–Dindo 4, n (%) [including fluid collection]	4 (12.5%)	4 (5.6%)	
Clavien–Dindo 5, *n* (%)	0	0	
Median length of hospital stay, days (IQR)	9 (6–16)	7 (5–11)	0.022
Median length of hospital stay (after surgery), days (IQR)	8 (5–13)	6 (4–8)	0.002
Readmission, *n* (%)	2	4	0.998
Mortality	0	0	

## Data Availability

Data described in the manuscript, code book, and analytic code will be made available upon request pending application and approval at the Corresponding Author e-mail address.
